# Effect of a multimedia training programme for pain management on pain intensity and depression in patients with non-specific chronic back pain*

**DOI:** 10.17533/udea.iee.v40n1e13

**Published:** 2022-03-30

**Authors:** Maryam Shaygan, Azita Jaberi, Roghayyeh Firozian, Zahra Yazdani, Nahid Zarifsanaiey

**Affiliations:** 1 Associate Professor. Community Based Psychiatric Care Research Center, Shiraz University of Medical Sciences, Shiraz, Iran. Email: m2620.shaygan@gmail.com Shiraz University of Medical Sciences Shiraz Iran m2620.shaygan@gmail.com; 2 Assistant Professor. Community Based Psychiatric Care Research Center. Shiraz University of Medical Sciences, Shiraz, Iran. Email: azita635@yahoo.com. Shiraz University of Medical Sciences Shiraz Iran azita635@yahoo.com; 3 Nurse, Student Research Committee, Shiraz University of medical sciences, Shiraz, Iran. E-mail: firoozianelahe@gmail.com Shiraz University of medical sciences Shiraz Iran firoozianelahe@gmail.com; 4 Nurse Educator. Department of Nursing, School of Nursing and Midwifery, Shiraz University of Medical Sciences, Shiraz, Iran. Email: researchnu1397@gmail.com Department of Nursing School of Nursing and Midwifery Shiraz University of Medical Sciences Shiraz Iran researchnu1397@gmail.com; 5 Associate Professor. Department of eLearning planning in Medical Sciences, Center of excellence for electronic learning, Shiraz University of Medical Sciences, Shiraz, Iran. Email: sanaieyn@sums.ac.ir Department of eLearning planning in Medical Sciences Center of excellence for electronic learning Shiraz University of Medical Sciences Shiraz Iran sanaieyn@sums.ac.ir

**Keywords:** pain management, low back pain, depression., manejo del dolor, dolor de la región lumbar, depresión., manejo da dor, dor lombar, depressão.

## Abstract

**Objective.:**

To determine the effect of multimedia training on pain intensity and depression in patients with chronic low back pain.

**Methods.:**

In this randomized controlled trial study, the intervention group was trained about pain management consisted of communication skills, assertiveness, stress management, lifestyle enhancement skills and physical activity prepared in seven CDs using multimedia method and the control group received routine training included physician’s visits, medication prescriptions and receiving the recommendations of the physician and healthcare providers. Beck Depression Inventory and Jensen Pain Questionnaire were completed for the two groups in three stages: pre-training, post-training and 2 months thereafter.

**Results.:**

The results showed that there were no significant statistical difference between two groups in terms of demographic variables indicated homogeneity of research groups. Repeated measure ANOVA showed that the mean scores of pain intensity and depression changed significantly over time in both control and intervention groups (*p*<0.001); however, the effect of the group was not significant (*p*=0.565, *p*=0.748, respectively). Hence, the results of time-group interaction showed that there was significant difference between the two groups in terms of pain intensity and depression (*p*<0.001, *p*=0.003, respectively). The effect size revealed that the difference between mean scores of depression before and after the intervention in the both group was high (1.04 and 1.45, respectively).

**Conclusion.:**

The study results indicated that multimedia training has the potential in relieving pain intensity and depression in patients with non-specific chronic low back pain.

## Resumen

**Objetivo.:**

Determinar el efecto del entrenamiento con multimedia sobre la intensidad del dolor y la depresión en pacientes con lumbalgia crónica.

**Métodos.:**

Ensayo clínico controlado y aleatorizado: el grupo de intervención recibió educación sobre el manejo del dolor consistente en unos temas (habilidades de comunicación, asertividad, manejo del estrés, habilidades para mejorar el estilo de vida y actividad física) disponibles en siete CD mediante el método multimedia; y el grupo de control recibió la educación rutinaria brindada durante las visitas al médico, consistente en la prescripción de medicamentos y la recepción de las recomendaciones del médico y de otros profesionales sanitarios. El Inventario de Depresión de Beck y el Cuestionario de Dolor de Jensen se aplicaron a los dos grupos en tres momentos: antes del entrenamiento, después del entrenamiento y dos meses después de acabar la intervención.

**Resultados.:**

El ANOVA de medidas repetidas mostró que las puntuaciones medias de la intensidad del dolor y de la depresión cambiaron significativamente a lo largo del tiempo tanto en el grupo de control como en el de intervención (*p*<0.001); sin embargo, el efecto del grupo no fue significativo (*p*=0.565, *p*=0.748, respectivamente). Por lo tanto, los resultados de la interacción tiempo-grupo mostraron que sí había una diferencia significativa entre los dos grupos en cuanto a la intensidad del dolor y la depresión (*p*<0.001, *p*=0.003, respectivamente). El tamaño del efecto reveló que la diferencia entre las puntuaciones medias de depresión antes y después de la intervención en ambos grupos era alta (1.04 y 1.45, respectivamente).

**Conclusión.:**

Los resultados del estudio indicaron que el entrenamiento multimedia tiene potencial para aliviar la intensidad del dolor y la depresión en pacientes con lumbalgia crónica inespecífica.

## Resumo

**Objetivo.:**

Determinar o efeito do treinamento com multimídia sobre a intensidade da dor e a depressão em pacientes com lombalgia crónica.

**Métodos.:**

Ensaio clínico controlado e aleatório, o grupo de intervenção recebeu educação sobre o manejo da dor consistente nuns temas (habilidades de comunicação, assertividade, manejo do estresse, habilidades para melhorar o estilo de vida e atividade física) disponíveis em sete CD mediante o método multimídia; e o grupo de controle recebeu a educação rotineira brindada durante as visitas ao médico, consistente na prescrição de medicamentos e a recepção das recomendações do médico e de outros profissionais sanitários. O Inventário de Depressão de Beck e o Questionário de Dor de Jensen se aplicaram aos dois grupos em três momentos: antes do treinamento, depois do treinamento e dois meses depois de acabar a intervenção.

**Resultados.:**

O ANOVA de medidas repetidas mostrou que as pontuações médias da intensidade da dor e da depressão mudaram significativamente ao longo do tempo tanto no grupo de controle como no de intervenção (*p*<0.001); porém, o efeito do grupo não foi significativo (*p*=0.565, *p*=0.748, respectivamente). Por tanto, os resultados da interação tempo-grupo mostraram que se havia uma diferença significativa entre os dois grupos enquanto à intensidade da dor e a depressão (*p*<0.001, *p*=0.003, respectivamente). O tamanho do efeito revelou que a diferença entre as pontuações médias de depressão antes e depois da intervenção em ambos os grupos era alta (1.04 e 1.45, respectivamente).

**Conclusão.:**

Os resultados do estudo indicaram que o treinamento multimídia tem potencial para aliviar a intensidade da dor e a depressão em pacientes com lombalgia crónica inespecífica

## Introduction

Chronic pain is one of the most important medical problems all around the world(1) contributed to disability, impaired quality of life, general health, and daily functioning, which in turn leads to the economic effects of using health services and unemployment hours.(2) Low back pain (LBP), a common type of chronic pain, is also accompanied with various consequences in physical and psychological health, and imposes financial burdens on families and healthcare system.(3) As a result, pain management is an essential aspect of patient care. On the other hand, it is well documented that there is an association between chronic pain and depression (4), with the prevalence rate of 33.9%.(5) Patients with LBP and depressive symptoms may be at risk of poorer recovery from LBP, poorer treatment outcomes or more health care utilization.(6) Therefore, it is important to consider pain management programs in order to reduce the depressive symptoms and consequent outcomes.

Clinical guidelines for chronic pain management suggest a multidisciplinary approach which includes a self-management framework with cognitive behavioral techniques, graded activities and exposure, skills training, education, and physical exercise.(7) Moreover, research investigated psychological factors of pain management programs outcome, such as depression, in some cases yielded contradictory findings.(8) Stress and anger management and assertiveness skills could reduce the stress in interpersonal communication and increase the relationships, which could eliminate the focus on pain. Besides, positive thinking skill would change the negative interpretation about pain and as a result would reduce the pain and depression in these patients.(9) It is noteworthy to mention that most pain management programs are implementing face-to-face. However, these programs generally require participants to engage in face-to-face sessions of moderate (30-60 hrs) to high (>60 hours) intensity and are mostly based in urban outpatient treatment facilities that restricts access for those living in rural areas.(7) Distance education methods such as multimedia in health were introduced as a solution for these kind of situations,(10) which can be performed via multimedia or internet-based methods.

Several Internet-based treatment programs for chronic pain have been developed, indicating efficacy in reducing disability, mood disturbance, and improving perceived self-efficacy.(11) However, the efficacy of these programs is variable due to methodological inconsistencies in the literature, including different outcome measures, key program content, types of control groups used, and clinician involvement.(12) Given the typically small to moderate effect sizes reported of Internet-based pain management programs, it is warranted to explore the viability of developing a novel program to improve treatment efficacy, such as multimedia methods.

Nonetheless, the studies conducted on multimedia methods for pain management are scarce. In a study, an interactive website along with multimedia eHealth pain management training resulted in decreasing the number of referrals to neurologists (13). In another study that interactive website, video messages and social media used for pain management campaign, the participants stated that if the healthcare providers could provide more information about this campaign, these interventions would be more useful.(14) The results of these studies shows that the multimedia could also use as a method of pain management training. Hence, there is limited information in this area in the culture of Asian countries, particularly Iran. Therefore, studies in different contexts and cultures are needed to provide more reliable results regarding the effects of pain management training on chronic LBP.(15) Furthermore, given the different environmental and treatment contexts, as well as the different types of pain and asso ciated challenges for those with chronic pain, there is unlikely to be a single self-management method or strategy for all occasions or all people.(16) Considering the paucity of literature related to the multimedia training for pain management and in the light of aforementioned information, the present study was performed to examine the effect of pain management training on pain intensity and depression in patients with chronic nonspecific low back pain.

## Methods

*Design, settings, participants, eligibility criteria.* This was a randomized controlled trial study with one experimental group (multimedia training group) and one control group and pre-test and post-test design conducted on patients with chronic low back pain referred to educational clinics affiliated to Fasa University of Medical Sciences from November 2018 to June 2019. Inclusion criteria included: Age over 18 years old, willingness to participate in research, chronic low back pain based on expert diagnosis according to physical examinations and diagnostic tests such as MRI, CT scan and imaging, having depression scores higher than 14 according to the Beck Depression Inventory. Exclusion criteria were: The reluctance or lack of possibility of continuing to participate in the research for any reason (such as the severity of the patient's pain), other chronic pains such as chronic headache, chronic psychiatric disorders such as schizophrenia, participation in similar training classes, lack of participation in more than two training sessions.

*Sample size calculation.* Sample size was calculated using the mean difference formula and the findings of a former study reporting that the α=0.05, β=0.2, (µ_1_-µ_2_)=4 and S=4.49.(17) Considering the 10% attrition rate, a sample size of 66 was selected as 33 for each group. Convenience sampling method was used and then qualified patients were randomly assigned to multimedia training group and control group using the random number table.

*Intervention.* Two education methods were designed for pain management, namely multimedia and the routine care. Educational materials for multimedia groups were developed using pain management training-related resource.(18) In previous studies, pain management training has included different physical, psychological and behavioral components, i.e. communication skills, assertiveness, stress management, lifestyle enhancement skills and physical activity.(19) Educational materials for topics were prepared as PowerPoint presentation slides in Shareable Content Object Reference Model (SCORM) format, then scenarios and audio explanations about educational materials were added to each slide. Accordingly, seven sets of educational materials were prepared in seven CDs for seven educational sessions. Each week, one CD was provided to participants in the multimedia group and they were asked to watch it at home. After two weeks and watching two CDs, during a face to face meeting, contents of the CDs were reviewed, the patients were encouraged to use the educational contents and then an educational pamphlet was given to them. Moreover, a WhatsApp group was formed where the second author answered their questions and encouraged them for using educational materials. The contents of the CDs were presented were: *Session 1-* Training acute and chronic pain difference and how the psychological factors affect the experience of chronic pain; *Session 2-* Training appropriate sports exercises*; Session 3-* Effective communication skill*; Session 4-* Training assertiveness skill*; Session 5-* Training stress management; *Session 6-* Positive thinking skill; and, *Session 7-* Anger coping skill. Participants in the control group received routine care services. In these centers, patients with chronic low back pain refer to receive medical services. These routine services include physician’s visits, medication prescriptions and receiving the recommendations of the physician and healthcare providers. At the end of the study, educational CDs were provided to participants in the control group and their questions were answered.

*Measures.* In this research, questionnaires for demographic characteristics, pain intensity, and depression were completed in three stages of pre-test (immediately before training) and post-test (after completing the training course) and one month after the end of the educational intervention during a visit by the researcher according to the time scheduled with the patient. The pain intensity was measured by Jensen's questionnaire. This questionnaire was developed by Jensen et al. in 2000. It is widely used in most studies related to pain, and it has reliability in all types of pain and populations. In the numerical scale method, pain is scored between 0-10 which the left side of the scale is numbered as no pain and right side as the most severe pain.(20) Results of previous studies confirmed the test-retest reliability, convergent validity (with other pain scales such as Visual Rating Scale) and discriminant validity.(21^,^^22^) Depression symptoms were measured by the short version of the inventory developed by Beck *et al*. known as Beck Depression Inventory-II (II-BDI). Similar to the first version, this inventory is composed of 21 items and each of which is scored between 0 -3 based on the intensity level reported by the patient. Scores of 0-13 indicate minimal depression, scores of 14-19 denote mild depression, 20-28 as moderate and 29-63 as severe depression.(23) According to Beck, Steer, and Garier, the second version also indicates the presence and intensity of depression symptoms in patients and normal population. They reported the internal consistency of this version as 0.73-0.92 with an average of 0.86 and an alpha coefficient of 0.86 for patients and 0.81 for non-patients.(24) The psychometric properties of this inventory in a sample of 94 people in Iran were confirmed by calculating the alpha coefficient of 0.91. The coefficient of correlation between the two half-tests was 0.89, and the re-test coefficient was 94.4.(25)

*Data analysis.* Data were analyzed at descriptive and inferential levels. To describe nominal and ordinal data, frequency distribution, number, and percent, and for interval and ratio data, descriptive, mean, and standard deviation were used. In inferential statistics, Chi-square, pair-wise t-test, ANOVA, and Pearson correlation coefficient were used, and repeated measurement tests were used to test the hypotheses. Data were analyzed by SPSS-22 software at the significance level of 0.05.

*Ethical considerations.* After being confirmed by the Ethics Committee of Shiraz University of Medical Sciences (IR.SUMS.REC.1396.117) and obtaining the approval of the authorities, the researchers attended in study settings. Afterwards, they asked the patients who were willing to take part in the study and took their consent. The study participants were fully aware of the study objectives and methodology. Maintaining the participants’ anonymity and data confidentiality, and the ability to leave the study at any stage were other ethical considerations. IRCT registration code: IRCT20180313039074N1.

## Results

The results showed that most of participants in the intervention group were female (83.3%), had academic education (40%), married (66.67%), with a mean age of 47.4 ± 7.5. In control group, most of them were female (66.7%), had academic education (50%), married (83.3%) with mean age of 53.2±12.6. No significant statistical difference was observed between two groups in terms of demographic variables such as age, sex, marital status, educational level, job, and duration of low back pain, which indicated homogeneity of research groups (Table 1).

**Table 1:** Demographic characteristics of the participants.

Categories	**Control (*n*=30)**	**Multimedia (*n*=30)**	***p*-value**
Age; mean±SD	53.2±12.6	51±9.7	0.458
Duration of back pain; mean±SD	8.6±5.2	8.23±6	0.802
Sex; *n* (%)			
Female	20 (66.7)	25 (83.3)	0.233
Male	10 (33.3)	5 (16.7)	0.233
Education; *n* (%)			
Primary/secondary education	5 (16.7)	7 (23.3)	0.7
High school certificate	10 (33.3)	11 (36.7)	0.7
College or university degree	15 (50)	12 (40)	0.7
Marriage status; *n* (%)			
Single	2 (6.7)	4 (13.33)	0.329
Married	25 (83.3)	20 (66.67)	0.329
Divorce/ Widowed	3 (3.3)	6 (20)	0.329
Employment; *n* (%)			
Housewife	11 (36.7)	12 (40)	0.926
Employed	14 (46.7)	14 (46.67)	0.926
Retired	5 (16.7)	4 (13.33)	0.926

Repeated measure ANOVA showed that participants' pain intensity and depression changed significantly over time in both control and intervention groups (*p*<0.001). Regarding the differences between the groups, the results showed that regardless of the time, the groups were not significantly different from each other, that is, in each of the 3 time periods, the two groups were not significantly different in terms of pain intensity and depression (*p*=0.565, *p*=0.748, respectively). Regarding the interaction of time and group, the findings indicated that the two groups were significantly different, which means the intervention in the multimedia group caused significant changes compared to the control group (*p*<0.001, *p*=0.003, respectively) (Table 2). However, before the intervention, the mean of depression scores in intervention group was higher than control group. The results of ANCOVA also showed that in assessing the effect of demographic data and the group simultaneously, there was no significant difference between two groups regarding pain intensity and depression. Moreover, to clarify the actual effect of the intervention on pain and depression, the effect size was calculated, which revealed that the difference between mean scores of depression before and after the intervention in the both group was high (1.04 and 1.45, respectively) (Table 2).

**Table 2:** Results of repeated measurement ANOVA for Pain intensity and depression

Measure	Pain intensity	Pain intensity	Depression	Depression
	Control	Intervention	Control	Intervention
Pretest	4.9±2.19	5.43±2.16	15.63±4.26	17.73±6.04
Post test	5.13±1.96	4.37±1.93	10.3±5.8	9.03±5.91
Follow up	4.97±1.94	4.1±1.65	9.73±6.2	7.83±5.26
*p*-value (Time effect)	<0.001	<0.001	<0.001	<0.001
*p*-value (Group effect)	0.565	0.565	0.748	0.748
*p*-value (Time*group interaction)	<0.001	<0.001	0.003	0.003
Pre-post effect size	0.11	0.52	1.04	1.45
Pre-follow up effect size	0.08	0.15	0.09	0.21

## Discussion

Current research was conducted aiming at investigating the impact of the multimedia training of pain management on pain intensity and depression in patients with non-specific chronic LBP. Concerning the research objectives, the results of the study indicated that multimedia training has the potential in relieving pain intensity and depression in patients with non-specific chronic low back pain. The results depicted that the mean scores of pain intensity and depression changed significantly over time in both control and intervention groups; however, the effect of the group was not significant. Hence, the results of time-group interaction showed that there was significant difference between the two groups in terms of pain intensity and depression.

The results of the present study are consistent with some other research. For example internet-based education improved the pain intensity in chronic back pain.(26) In some other studies multimedia pain management training has also been found to be effective as a mHealth method.(14) To explain the effect of multimedia education on pain and depression, the content of CDs might be the key point. It is well established in previous literature that social factors have been linked to the etiol ogy and maintenance of chronic pain. One salient social fac tor is early life stress. As a result of social challenges, individuals may face elevated risks of chronic physical pain due to a variety of factors, including an insecure styles of interpersonal attachment. Another socially based nega tive emotional state that has been found to be a predictive of later pain intensity is daily ratings of loneliness. Moreover, individ uals with chronic pain tend to appraise their social relationships more negatively when experiencing elevated levels of stress or negative emotion.(9) These findings suggest that both the presence of social conflict and the evaluation of one’s social rela tionships have implications for emotional states in chronic pain. Consistently, Sturgeon *et al*. also believed that encouraging adaptive interpersonal communication along with increasing adaptive behaviors could help patients to cope with chronic pain.(27) All aforementioned studies emphasized the inclusion of stress management and interpersonal communication skills in pain management training,(28) as we did in present study. On the other hand, face to face meeting every two weeks, answering the participants' questions, encouraging them to use the methods explained in CDs and providing educational pamphlets helped the consolidation of learning. This is in line with the opinion of the educational investigators about "blended learning". They believe that distance education methods are not enough for learning and should be combined with face to face methods and feedback and reflection about teaching/learning should be performed.(29)

To explain the elimination of depression, it could be said that the causative agent for depression is not just the pain experience, but psycho-social factors and especially cognitive/believe thought factors are also effective. Negative thoughts about pain, interpreting pain as a mysterious and non-understandable phenomenon, and not having control over pain feeling are some of these factors.(30) Although there was no intervention in the control group, the mean scores of depression also decreased in this group. However, this decrease was greater in the multimedia group. Regarding this, it can be said that education was not the only factor in reducing depression and depression was not only a result of pain in patients and other factors were involved. Inaccuracy and attention of patients in answering questionnaire questions can also be one of the causes of decrease in depression score after intervention. Another limitation of this study was the exclusive reliance on self-reports for the quantitative data. It is an assumption that people would be as honest as possible to the extent that they were aware of their own thoughts, feelings, and functional abilities at the time of the data collection. Nonetheless, some patients with chronic pain may either exaggerate or minimize their reports of pain.

Although based on the results of this study, demographic factors such as age, gender, education, marriage status and job did not have a confounding effect on pain and depression, there might be other factors not addressed in this study such as other stressors in life and job, using specific drugs with depressive side effects and sleep disorders as mediating factors. As such, studying the effect of these factors should be warranted in the future studies.

## Conclusion

The positive effect of multimedia pain management training on pain intensity and depression level in patients with chronic low back pain indicates that these methods could help to reduce the pain and suffering of them. Also, considering non-medical methods in the treatment of these patients, in addition to reducing pain, would help reduce their depression. Nurses and other health team members who care for these patients could help alleviate this agony using these methods. Future studies into chronic LBP management are recommended to assess the effects of pain management training on different aspects of pain such as pain-related interference, pain tolerance, and quality of life. Moreover, studies with similar pain intensity and depression level assessment methods are recommended to produce comparable results.
